# Matters of development and experience: Evaluation of dog and human emotional expressions by children and adults

**DOI:** 10.1371/journal.pone.0288137

**Published:** 2023-07-26

**Authors:** Heini Törnqvist, Hanna Höller, Kerstin Vsetecka, Stefanie Hoehl, Miiamaaria V. Kujala

**Affiliations:** 1 Department of Psychology, University of Jyväskylä, Jyväskylä, Finland; 2 Department of Developmental and Educational Psychology, University of Vienna, Vienna, Austria; 3 Faculty of Veterinary Medicine, University of Helsinki, Helsinki, Finland; 4 Department of Neuroscience and Biomedical Engineering, Aalto University, Espoo, Finland; Università degli Studi di Bari: Universita degli Studi di Bari Aldo Moro, ITALY

## Abstract

Emotional facial expressions are an important part of across species social communication, yet the factors affecting human recognition of dog emotions have received limited attention. Here, we characterize the recognition and evaluation of dog and human emotional facial expressions by 4-and 6-year-old children and adult participants, as well as the effect of dog experience in emotion recognition. Participants rated the happiness, anger, valence, and arousal from happy, aggressive, and neutral facial images of dogs and humans. Both respondent age and experience influenced the dog emotion recognition and ratings. Aggressive dog faces were rated more often correctly by adults than 4-year-olds regardless of dog experience, whereas the 6-year-olds’ and adults’ performances did not differ. Happy human and dog expressions were recognized equally by all groups. Children rated aggressive dogs as more positive and lower in arousal than adults, and participants without dog experience rated aggressive dogs as more positive than those with dog experience. Children also rated aggressive dogs as more positive and lower in arousal than aggressive humans. The results confirm that recognition of dog emotions, especially aggression, increases with age, which can be related to general dog experience and brain structure maturation involved in facial emotion recognition.

## Introduction

Interpreting emotions from facial expressions is an important part of nonverbal social communication across species and lifespan [1–3]. Already Darwin [4] claimed that emotions are expressed by all species and that humans can determine other animals’ emotions from their facial and bodily expressions [but see also 5]. Several brain areas have been found to be involved in the processing of facial emotions, but studies are still lacking consensus and there has been variability in study results [6, 7]. Multiple variables can impact to the recognition of facial expressions, for example respondent’s gender and partially occluding the face area [8, 9]. Humans are experts in recognizing another person’s emotions by observing facial expressions [e.g., 10–12], but humans’ ability to recognize dog emotional expressions has been studied less [13]. Only a few studies have investigated the ability of young children to recognize and evaluate dogs’ facial expressions or made comparisons between the abilities of children and adults [14–16]. Previous studies suggest that young children lack abilities to interpret emotional expressions of dogs, which deserve more detailed attention in order to improve the quality and understanding of the real-life interactions between children and dogs.

Previously, dog owners’ attribution of emotions to their dogs have been commonly investigated by rating discrete emotions, such as happiness and aggressiveness [17, 18]. However, little research has used valence and arousal ratings of dog emotions based on two-dimensional approach, where emotions can be described using negative/positive valence dimension and low/high arousal dimension [19–21]. To our knowledge, this is one of the first studies, where valence and arousal ratings were combined with rating of discrete emotion to assess children’s abilities to interpret dog emotions from facial expressions [see also 15].

In previous studies, the human evaluation of dog emotional signals has been investigated with auditory dog bark playback stimuli [22, 23], with audio-visual dog vocalization and behavior recordings [16, 24], with dog facial images [13, 14, 25] and/or full body videos of dogs [15, 26–29]. Long-shared history and the special human-dog bond may have led both humans and dogs to develop skills to understand each other’s emotional expressions [e.g., 13, 22, 30–33]. According to previous studies, pleasant and threatening dog facial expressions are classified in a similar manner as human facial expressions [25, 34]. However, all emotions are not expressed alike across species, and for humans, some dog emotions might be easier to recognize than others. For example, dogs’ playful/happy expressions appear easier to recognize than aggressiveness or fear [13, 35–37].

Also, the effect of human everyday experience with dogs (e.g., having a dog in the family) in recognizing dog emotions is still unclear. Co-domestication hypothesis suggests that humans have the ability to read dog emotions through emotionally adapted mechanisms, meaning that these skills should be partially present also without dog experience [e.g., 38, 39, but see 35]. In some of the previous experiments, humans without dog experience have been as good as humans with dog experience in recognizing dog emotions from auditory or visual cues [22, 34] or sometimes even better [13, 28], whereas in other experiments, dog experience has improved dog emotion recognition especially in adults [14, 40]. Additionally, dog expertise was also found to affect both neural processing of and visual attention to dog social interaction in adult observers in a brain imaging study [41]. As expertise effects in interpretation of dogs has been found in a number of studies, it is thus also possible that dog experience influences perception and recognition of dog emotional expressions as emotion recognition is an important part of social communication. One explanation for the apparent controversy of the previous results is differing levels of experience (from having a dog in the family to experienced dog trainer) used in different experiments, together with other possible affecting factors such as different types of stimuli used (e.g., visual, auditory or both). Therefore, more research is needed to disentangle the possibly multiple concurrent sources of the factors affecting the human perception of canine emotions.

Facial expressions of emotions are processed in a large network of brain areas including prefrontal cortices, the fusiform gyrus, insula, and the amygdala among others [e.g., 42–47]. Studies suggest that these brain structures continue to mature throughout late childhood to adolescence, and that recognition of facial emotions may not reach maturity before adult age [e.g., 3, 48, 49]. Also, Theory of Mind (ToM), which is the ability to ascribe mental states to others, continues to develop beyond preschool years [50; for a review e.g., 51, 52]. A previous study showed that 4–5-year-old children have limited ability to understand dog bodily signals compared to 6–12-year-old children [16]. Limited ability in reading dogs’ body postures and facial expressions can lead into dangerous situations [e.g., 29, 53]. For example, children may assume that a dog with exposed teeth is smiling although the expression is a serious warning signal to keep distance [e.g., 16, 54]. Another study reported that 5–6-year-old children were less adept than adults at reading dog emotions, except for anger and happiness that were also recognized by children [14]. Thus, the literature suggests effects of human developmental stage in the evaluation of dog emotional expressions, but the time courses of development and exact effects appear somewhat unclear.

Here, we investigated how different dog and human emotional expressions are recognized and evaluated by 4-and 6-year-old children and adults. These age groups were selected on the basis of previous literature: 3–5-year-old children have shown limited ability to read dogs’ emotions compared to older children and adults [e.g., 16, 24, 36]. We also wanted to further characterize the effect of daily dog experience, or exposure (i.e., whether there has been dog in the family) in recognizing dog expressions, henceforth referred as “dog experience”. The first aim of this study was to evaluate the effect of age on the emotion detection, and also quantify whether the age has an effect on valence and arousal ratings. Based on previous studies [e.g., 15, 16, 29], we expected that the performance of 4-year-olds, 6-year-olds and adults differ from each other. The second aim was to assess the effect of daily dog experience on dog emotion recognition. Based on previous studies [14, 40], we assumed that dog experience has an effect especially on adults’ performance, because of their cumulative experience with dogs. The third aim was to clarify how children and adults recognize and rate affect in dog facial expressions compared to human expressions. In previous literature, negative and positive expressions in humans and dogs have been processed quite similarly by children and adults [14, 25, 34], but also some differences have been found in the ability of children to recognize dog emotions compared to human emotions [14, 15].

## Materials and methods

### Ethics statement

The adult study had been previously approved by the Aalto University ethics committee (board meeting held 06/03/2014). Prior to the experiment, participants gave their written informed consent. The child study was approved by the Ethics Committee of the University of Vienna (reference number 00557). Children’s guardians provided an informed consent and children also provided verbal consent before starting the experiment. Both studies with adults and children were conducted according to the Declaration of Helsinki.

### Participants

Altogether 34 healthy volunteers (15 males and 19 females; mean age 36.9 years; range 25 to 46 years) participated in the adult study and 26/34 of the participants had had a dog in the family. Adult participants were recruited from the university networks, mailing lists and through social media. The data of adults was originally collected for Kujala et al. [25] and re-analyzed in this project with a different aim. Recruiting and testing with adults was conducted from March 2014 to July 2014. Additionally, in total 28 children (13 boys and 15 girls) aged 4 years (mean age 4.5; range (years; months) 4;0–5;5) and 31 children (12 boys and 19 girls) aged 6 years (mean age 6.3; range 5;10–7;2) participated in the children study. The number of children that had had dog in the family was 10/28 of the 4-year-olds and 12/31 of the 6-year-olds. Children were recruited through social media ads and the laboratory’s existing database of families interested in participating in developmental research. Children’s recruiting and testing was conducted from October 2020 to October 2021. In both studies, data anonymization was used, and authors had no access to information that could identify individual participants during or after data collection.

### Stimuli

Stimuli for the adult and children study consisted altogether of 48 color images of dog and human faces. Originally, adults were also shown additional images, which were part of another study with different aims [25] and not included in the current experiment. The dog face stimuli were selected by experienced dog trainer and facial action coding systems (FACS and dog-FACS) were used to characterize the human and dog facial expressions [55, 56]. The stimuli were pre-labeled by the facial expressions and species as Happy Dogs (relaxed, mouth open, tongue visible), Aggressive Dogs (showing teeth), Neutral Dogs (mouth closed, expressionless), Happy Humans (smiling, teeth visible), Aggressive Humans (mouth open, teeth visible) and Neutral Humans (mouth closed, expressionless), 8 images per category [25, 32]. For both adults and children, half of the actors in the images were male and half of them were female; faces were unfamiliar to participants; and images were cropped to contain only the face (and fur that was around the dog face) and ear area. Dog images consisted of many different breeds and mongrels. The human and dog facial images were obtained from online royalty-free databases (BigStockTM and 123RF^®^) and some of the dog facial images were obtained from a photographer Aino Pikkusaari. For additional information and samples of the human and dog face stimuli, see Somppi et al. [32].

### Experimental procedure

In adults, stimuli were presented with Presentation^®^ software on a laptop with 14-inch LCD screen. Images were overlaid on gray background and the size of the images was approximately 15 x 16 cm. The order of the images was pseudorandomized so that images in the same category were not presented more than three times subsequently. Each of the images was shown until the participant answered to all questions and the next image was presented right after.

In children, stimuli were presented with an experiment creating program called OpenSesame [57]. Facial images were overlaid on grey background and underneath the images, different scales were shown, one at the time, for answering the questions (the scales are described in detail in the next paragraph). Since the study had to be conducted online due to Covid-19 regulations at the time, the stimuli were presented on the subjects’ own laptops at home while communicating with the experimenter via JitsiMeet, a software for video conferencing. The stimuli were presented in a pseudorandomized order. Two stimulus sequences were created with the intermixed 48 stimuli: The first sequence was used for odd participant IDs, the second for even participant IDs. In the second sequence, the stimuli were presented in reverse order. When a participant had answered to all questions, the next image was presented right after.

### Rating of emotional expressions

The adults individually performed the experiment in an office room, and they were given information that different images of human and dog faces will be shown to them. Adults were instructed to view images freely and to evaluate the feeling and emotional state of the target in the image. Participants were also told that there were no correct answers and that we were interested in their subjective views and ratings. Before the actual experiment, a practice session was completed by rating four images not being part of the actual experiment. Participants verified the understanding of the task and procedure. For the duration of the experiment, the experimenter waited outside the room to provide the participant complete privacy for the task.

**Fig 1:**
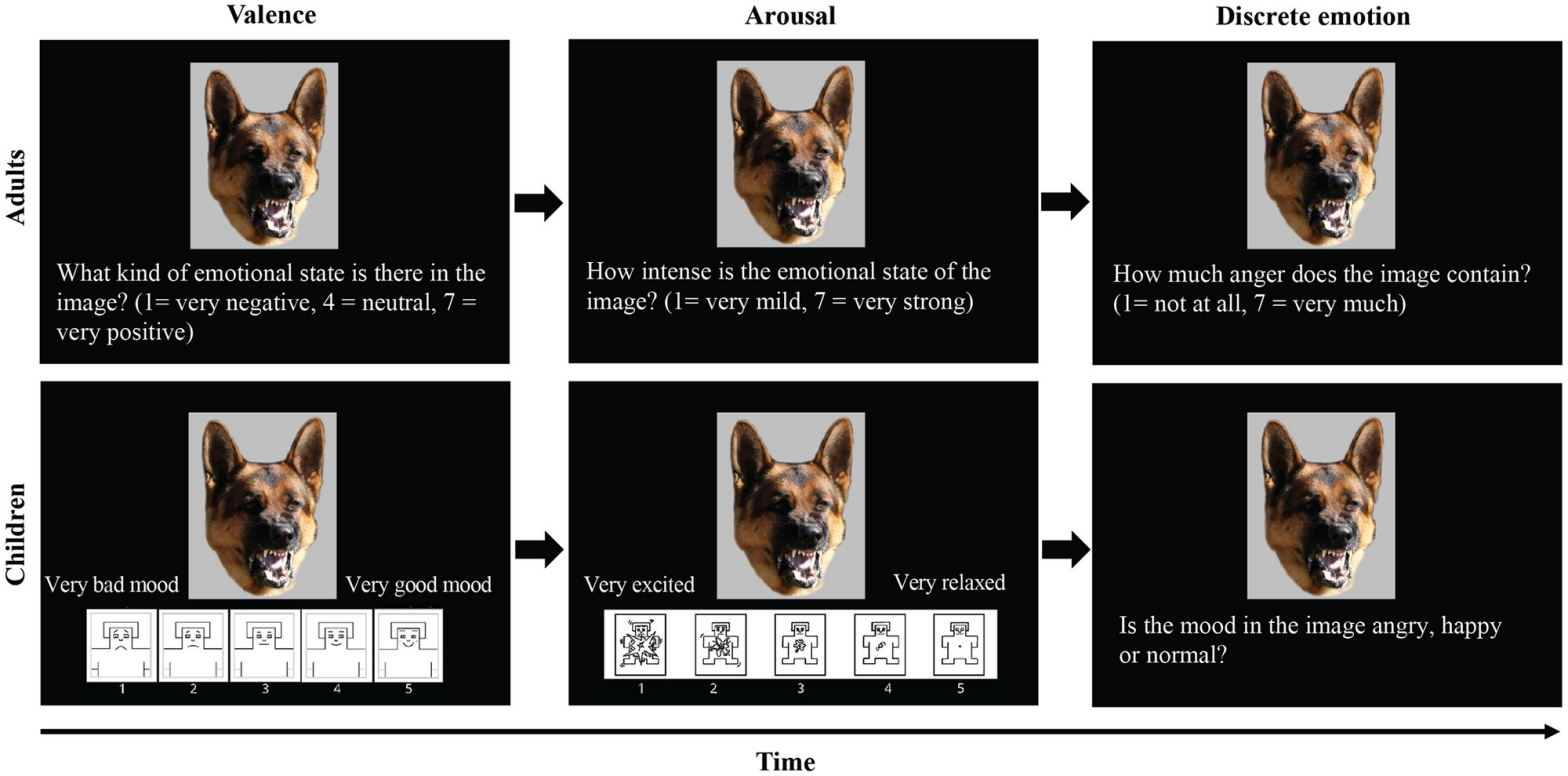
An example of a stimulus sequence presented to adults (first row) and children (second row). Participants were asked to rate valence (left column), arousal (middle column) and discrete emotions (right column) of the dog and human faces. Adults answered the questions by pressing the number buttons 1–7 on the keyboard and children selected the SAM figure from the scale by pointing it at the screen. For discrete emotions children answered by choosing between angry, happy, or normal/neutral and giving their answer verbally. There was no time limit in answering the questions, and once participant had answered the question, the following question was displayed. After answering all questions, next dog/human facial image was displayed. The dog image was purchased from 123RF with a license to publish this image in electronic and printed documents and reports. The SAM figures were reprinted from Bradley & Lang [58] with permission from Elsevier.

The adult participants answered questions that appeared at the bottom of the screen, one at a time, by pressing the number buttons 1 to 7 on the keyboard (Fig 1). There were eight different questions for each image. Once a question was answered, the following question was displayed on the screen. There was no time limit in answering the questions, but participants were encouraged to respond based on their initial impression. The first and second question sampled the valence (from very negative to very positive) and arousal (from no arousal to high arousal) of the images, which were evaluated on a scale 1–7 and had always the same order of presentation. In questions 3–8 the order was randomized, and the questions considered the basic emotions: happiness, anger/aggression, sadness, surprise, disgust, and fear. Participants were asked for example, “How much anger does the image contain?” (1 = not at all, 7 = very much). The six basic emotions were rated to obtain larger variety of possible emotions people could infer to the images. In this study, only happiness and aggressiveness ratings were included in the analyses for comparative purposes (see Fig 1). In general, it took 3 ± 1 s (mean ± SD) for participants to answer one question and about 0.5 to 1 hours to rate all the images. The entire experiment with instructions given in the beginning, practice session, rating the images and answering questionnaires lasted about 1.5 hours.

The children performed the experiment online with an accompanying person, communicating with the experimenter via videocall software. At the beginning of the experiment, the accompanying person alone was informed about the procedure and goals of the study and briefly instructed not to influence the child’s reactions and answers. In addition, the accompanying person was asked to follow along with their finger on the screen while the experimenter explained the individual scales to the child. Thereafter, the participating child also received instructions in child-friendly language about the procedure and how to answer the questions using different scales. The accompanying person and the child were also informed that breaks could be taken at any time and that the experiment could be stopped altogether without giving a specific reason.

After the instructions, children were asked to rate the dog and human faces according to their valence and arousal, as well as to assign a discrete emotion to the faces. With the help of the Self-Assessment Manikin Scale [SAM; 58], the valence and arousal ratings could be assessed in a child-friendly way. The scales each consist of five different figures, which are graded in their valence (negative to positive) and arousal (high to low), respectively. For the present study, only the head portion of the SAM figure was used from the valence scale in order to better identify the emotion of the figure. The children then selected the figure they felt matched best the emotion of the facial image presented by pointing at the screen. The figures were also numbered from one to five so that the accompanying person could verbally communicate the figure chosen by the child to the experimenter. The explanation of the SAM scale was briefly repeated after every eighth image during testing to prevent children from forgetting the meaning of each figure. The discrete emotion was asked via forced-choice format. This was done to reduce complexity and increase feasibility for children. The children could choose between angry/aggressive, happy, or neutral/normal and give their answer to the experimenter verbally (see Fig 1). The entire experiment lasted about 45 minutes.

### Questionnaires

After adults had rated the stimuli, they answered several questionnaires that were part of another study [25]. In this study, we targeted a question about participants’ overall dog experience (“Has there ever been a dog in the family?”), which was included in the analysis. The accompanying persons of the children also answered several questionnaires, and for this study, a question with two parts was chosen to represent children’s overall dog experience or exposure (“Have pets lived in the same household in the past (since the birth of your child)?”; “If yes, was it a dog?”).

### Data processing

To render the data from adults and children comparable, the rating scales were transformed for the data analysis. In the original emotion ratings, adults were asked “How much happiness/anger the image contains?” In which they answered in scale 1–7 (1 = not at all, 7 = very much). Children were asked “Do you think the dog/human is mostly angry, happy or in normal mood?” If the child’s answer was correct (e.g., there was an angry/aggressive dog in the image and child answered angry), it was coded as 1, and 0 if it was incorrect (e.g., there was an angry/aggressive dog in the image and child answered happy).

To be on the same scale with the child answers, adult answers from 1 to 4 with matching stimulus were transformed as incorrect answers (value 0) and answers from 5 to 7 as correct answers (value 1). For example, a happy dog or human image was presented, and an adult was asked how much happiness the image contains. If the value of participant’s answer was between 1–4 (i.e., representing answers from not at all to average), it was coded as incorrect (0) and if it was between 5–7 (i.e., representing answers from above average to very much), it was coded as correct (1). In the adult scale value 4 represented neutral, corresponding value 0 in children’s scale. The same was done for aggressive/angry dog and human faces when an adult was asked how much anger/aggressiveness the image contains. For the neutral dog and human faces, the adult was again asked how much happiness and anger/aggressiveness the image contains, and if the value of the participant’s answer to both happiness and anger/aggressiveness was 1 (i.e., representing answer not at all), it was coded as correct (1), otherwise as incorrect (0).

In the valence and arousal ratings, children answered the questions in scale -2 to 2 (5-point-scale), which was transformed into adults’ scale from 1 to 7 (7-point-scale) by using a formula x3 = 1.5*x2–0.5, where x2 = x1 + 3 (e.g., transforming data to a common scale, see www.ibm.com/support/pages/transforming-different-likert-scales-common-scale).

### Statistical analyses

The statistical analyses were carried out with SPSS statistics v. 28.0 (IBM, New York, NY, USA). The emotion, valence and arousal ratings of children and adults were compared with separate repeated measures ANOVAs with between-subject factors ‘age group’ (4 years, 6 years, and adults) and ‘dog experience’ (yes/no) and within-subjects factors ‘species’ (Dog, Human) and ‘emotion’ (Happy, Angry, Neutral). To clarify ANOVA results, a priori planned comparisons between the variables of interest were conducted with paired samples t-tests. To evaluate the robustness of the effect, the effect sizes are reported (partial eta squared for ANOVA results, and Cohen’s *d* for the planned comparisons).

## Results

### Recognition of discrete emotions

Age groups differed statistically significantly in their ratings of discrete emotions (between-subjects factor age group, F_2,87_ = 9.5, p<0.001, $\eta_{p}^{2}=0.18$, repeated-measures ANOVA). Also, main effects of species (F_1,87_ = 31.5, p<0.001, $\eta_{p}^{2}=0.27$) and emotion (F_2,167_ = 9.5, p<0.001, $\eta_{p}^{2}=0.10$) were found. A main effect of dog experience was not statistically significant (F_1,87_ = 0.0, p = 0.964, $\eta_{p}^{2}=0.00$), but there was a significant interaction effect between age group, emotion, and dog experience (F_4,167_ = 3.3, p<0.05, $\eta_{p}^{2}=0.07$). Clarifying the effect of species, (i.e., dog *vs*. human expressions), Aggressive Humans were rated more often correctly than Aggressive Dogs by 4-year-olds, regardless of dog experience (Without dog experience: 0.8 ± 0.1 vs. 0.4 ± 0.1, respectively; t_17_ = 3.3, p<0.01, *d* = 0.78; With dog experience: 0.9 ± 0.1 vs. 0.3 ± 0.2, respectively; t_9_ = 3.7, p<0.01, *d* = 1.16). The 6-year-olds without dog experience rated Aggressive Humans more often correctly than Aggressive Dogs (1.0 ± 0.1 vs. 0.5± 0.2, respectively; t_18_ = 3.6, p<0.01, *d* = 0.83), and there was no difference between ratings of Aggressive Humans and Dogs in 6-year-olds with dog experience (1.0 ± 0.0 vs. 0.8 ± 0.1, respectively; t_11_ = 1.5, p = 0.166, *d* = 0.43). In contrast, adults with dog experience rated Aggressive Dogs expressions more often correctly than Aggressive Humans (0.9 ± 0.1 vs. 0.5± 0.1, respectively; t_25_ = 4.0, p<0.01, *d* = 0.78). In addition, Happy Humans were rated more often correctly than Happy Dogs in adults with dog experience (0.9 ± 0.1 vs. 0.4± 0.1, respectively; t_25_ = 4.0, p<0.001, *d* = 0.79).

**Fig 2:**
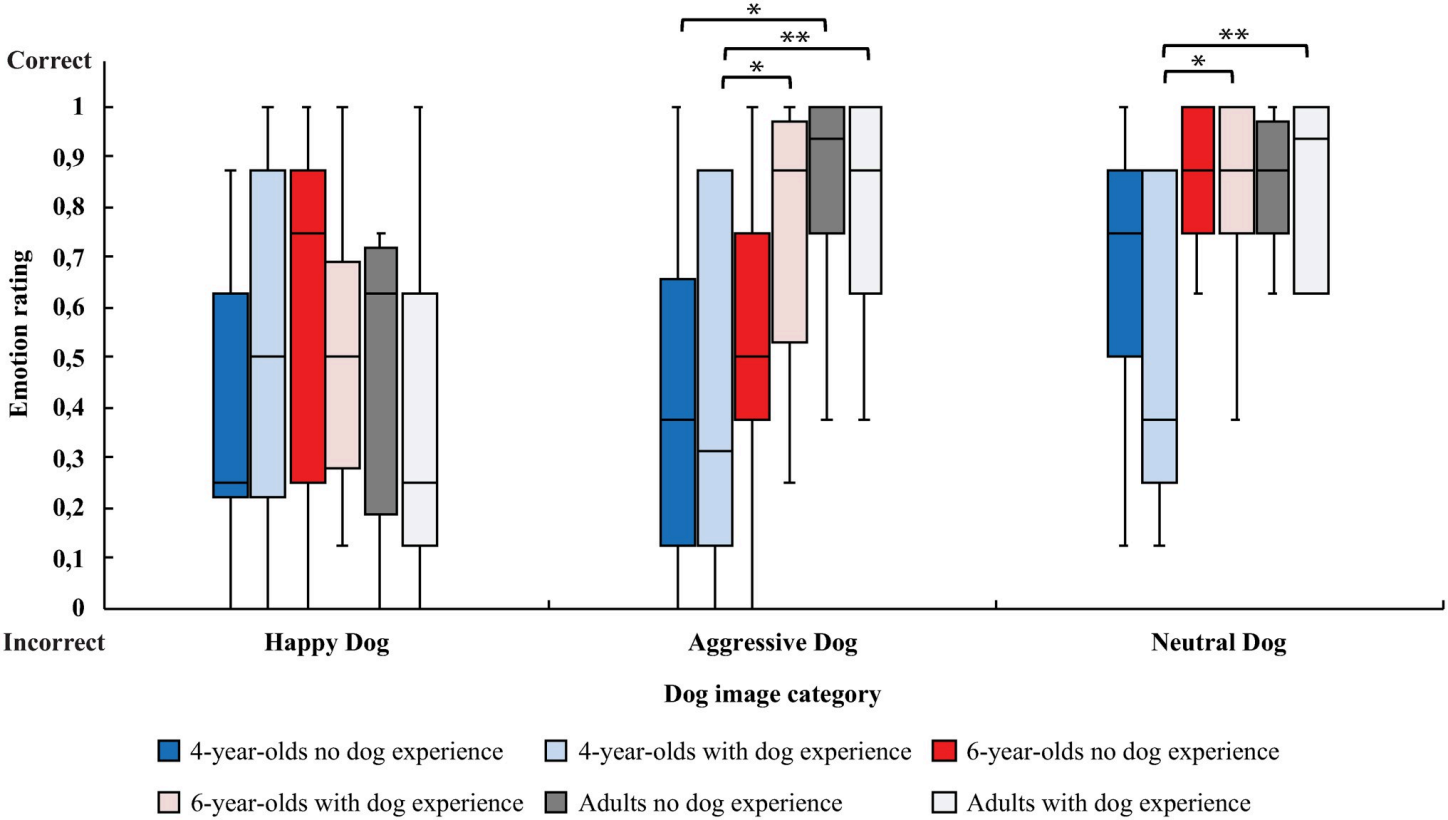
Emotion ratings for dog image categories (Happy Dog, Aggressive Dog, Neutral Dog) between age groups. Ratings are displayed separately for participants with experience of living in the same household with a dog (with dog experience) or without such experiences (no dog experience). Value 0 means that participants have answered incorrectly when asked what kind of emotion there is in the image and value 1 that they have answered correctly. Statistically significant differences between the participant groups are represented by asterisks (**p<0.01 and *p<0.05).

Clarifying the effect of age (i.e., between age groups 4 yo *vs*. 6 yo *vs*. adults; Fig 2), 6-year-old children with dog experience rated Aggressive and Neutral Dogs more often correctly than 4-year-old children with dog experience (Aggressive Dogs: 0.8 ±0.1 vs. 0.3 ± 0.2, t_17_ = 2.8, p<0.05, *d* = 1.2; Neutral Dogs: 0.9 ± 0.1 vs. 0.4 ± 0.2, t_14_ = 2.8, p<0.05, *d* = 1.3). Adults rated Aggressive Dogs more often correctly than 4-year-old children, regardless of dog experience (Without dog experience: 0.9 ± 0.1 vs. 0.4 ± 0.1, t_19_ = 2.5, p<0.05, *d* = 0.92; With dog experience: 0.9 ± 0.1 vs. 0.3 ± 0.2, t_11_ = 3.9, p<0.01, *d* = 1.8). In addition, adults with dog experience rated Neutral Dogs more often correctly than 4-year-old children with dog experience (1.0 ± 0.0 vs. 0.4 ± 0.2, t_9_ = 3.7, p<0.01, *d* = 2.26). There were no statistically significant differences between adults and 6-year-old children in dog emotion ratings. In human image ratings, adults rated Happy Humans more often correctly than 4-year-old children (0.9 ± 0.1 vs. 0.7 ± 0.1, t_43_ = 2.3, p<0.05, *d* = 0.61). In addition, adults rated Neutral Humans more often correctly than 4-year-old children (1.0 ± 0.0 vs. 0.8 ± 0.1, t_34_ = 2.2, p<0.05, *d* = 0.60). Aggressive Humans were rated more often correctly by 4-year-olds (0.9 ± 0.1 vs. 0.6 ± 0.1, t_59_ = 2.2, p<0.05, *d* = 0.55) and 6-year-olds (1.0 ± 0.0 vs. 0.6 ± 0.1, t_42_ = 3.9, p<0.001, *d* = 0.9) than adults. There were no statistically significant differences between 4-and 6-year-old children in human emotion ratings.

Significant interaction effects between age group and species (F_2,87_ = 4.6, p<0.05, $\eta_{p}^{2}=0.10$), emotion and species (F_2,149_ = 6.7, p<0.01, $\eta_{p}^{2}=0.07$) and age group, emotion, and species (F_3,149_ = 6.1, p<0.001, $\eta_{p}^{2}=0.12$) were found. Clarifying the ANOVA effects, the a priori planned comparisons showed that between human categories, Aggressive Humans were rated correctly more often than Happy Humans by 4-year-old and 6-year-old children (Table 1). Adults rated Happy and Neutral Humans correctly more often than Aggressive Humans. Between dog categories, 4-and 6-year-old children without dog experience rated Neutral Dogs correctly more often than Happy Dogs or Aggressive Dogs. Adults with dog experience rated Aggressive Dogs correctly more often than Happy Dogs. In addition, they rated Neutral Dogs correctly more often than Happy Dogs with and without dog experience.

**Table 1:** The differences in recognition of discrete emotions (scale 0–1) between human categories and between dog categories in 4-year-olds, 6-year-olds, and adults (Paired t-tests t- and p-values, and Cohen’s *d* effect sizes).

	Image category	4-year-olds				6-year-olds				Adults			
		mean (SEM)	t	p	*d*	mean (SEM)	t	p	*d*	mean (SEM)	t	p	*d*
Dog experience	Aggressive vs. Happy Human	0.86 (0.1) 0.68 (0.1)	2.4	0.022*	0.46	0.97 (0.1) 0.77 (0.1)	2.7	0.012*	0.48	0.62 (0.1) 0.91 (0.1)	3.7	0.001***	0.64
	Happy vs. Neutral Human	0.68 (0.1) 0.79 (0.1)	0.8	0.415	0.16	0.77 (0.1) 0.90 (0.1)	1.4	0.161	0.26	0.91 (0.1) 0.97 (0.1)	1.0	0.325	0.17
	Neutral vs. Aggressive Human	0.79 (0.1) 0.86 (0.1)	0.6	0.537	0.12	0.90 (0.1) 0.97 (0.1)	1.4	0.161	0.36	0.97 (0.1) 0.62 (0.1)	4.2	0.001***	0.73
With dog experience	Aggressive vs. Happy Dog	0.30 (0.2) 0.60 (0.2)	1.4	0.193	0.44	0.83 (0.1) 0.58 (0.1)	1.1	0.275	0.33	0.92 (0.1) 0.42 (0.1)	5.0	0.001***	0.98
	Happy vs. Neutral Dog	0.60 (0.2) 0.40 (0.2)	0.8	0.443	0.25	0.58 (0.1) 0.92 (0.1)	1.8	0.104	0.51	0.42 (0.1) 1.00 (0.0)	5.8	0.001***	1.15
	Neutral vs. Aggressive Dog	0.40 (0.2) 0.30 (0.2)	0.4	0.678	0.14	0.92 (0.1) 0.83 (0.1)	0.6	0.586	0.16	1.00 (0.0) 0.92 (0.1)	1.4	0.081	0.28
Without dog experience	Aggressive vs. Happy Dog	0.44 (0.1) 0.28 (0.1)	1.1	0.269	0.27	0.53 (0.1) 0.58 (0.1)	0.3	0.749	0.08	0.88 (0.1) 0.63 (0.2)	1.0	0.175	0.35
	Happy vs. Neutral Dog	0.28 (0.1) 0.83 (0.1)	3.3	0.004**	0.79	0.58 (0.1) 0.95 (0.1)	3.2	0.005**	0.74	0.63 (0.2) 1.00 (0.0)	2.0	0.040*	0.73
	Neutral vs. Aggressive Dog	0.83 (0.1) 0.44 (0.1)	2.7	0.015*	0.64	0.95 (0.1) 0.53 (0.1)	3.6	0.002**	0.83	1.00 (0.0) 0.88 (0.1)	1.0	0.175	0.35

* significant at p < 0.05,

**significant at p < 0.01,

***significant at p < 0.001

### Valence ratings

Main effects of age group (F_2,87_ = 21.0, p<0.001, $\eta_{p}^{2}=0.33$) and emotion (F_2,153_ = 376.5, p<0.001, $\eta_{p}^{2}=0.80$, repeated-measures ANOVA) were found in valence ratings. A main effect of dog experience was not statistically significant (F_1,87_ = 0.0, p = 0.966$\;\eta_{p}^{2}=0.00$), but significant interaction effects between emotion and dog experience (F_2,153_ = 3.6, p<0.05, $\eta_{p}^{2}=0.04$), age group, species, and dog experience (F_2,87_ = 3.5, p<0.05, $\eta_{p}^{2}=0.08$) and emotion, species, and dog experience (F_2,163_ = 4.9, p<0.05, $\eta_{p}^{2}=0.05$) were found. Overall, Aggressive Dogs were rated as more positive by participants that didn’t have dog experience than by participants that had dog experience (3.5 ± 0.2 vs. 2.4 ± 0.2, respectively; t_87_ = 3.9, p<0.001 *d* = 0.81). Between dog categories, when participants with and without dog experience were compared, all participants rated Happy Dogs as more positive than Neutral Dogs (With dog experience: 5.3 ± 0.1 vs. 4.5 ± 0.1, t_47_ = 6.6, p<0.001, *d* = 0.53; Without dog experience: 5.4 ± 0.1 vs. 4.3 ± 0.2, t_44_ = 6.1, p<0.001, *d* = 0.76) or Aggressive Dogs (With dog experience: 5.3 ± 0.1 vs. 2.4 ± 0.2, t_47_ = 13.7, p<0.001, *d* = 0.40; Without dog experience: 5.4 ± 0.1 vs. 3.5 ± 0.2, t_44_ = 9.5, p<0.001, *d* = 0.13). In addition, Neutral Dogs were rated as more positive than Aggressive Dogs (With dog experience: 4.5 ± 0.1 vs. 2.4 ± 0.2, t_47_ = 12.7, p<0.001, *d* = 0.18; Without dog experience: 4.3 ± 0.2 vs. 3.5 ± 0.2, t_44_ = 3.3, p<0.001, *d* = 0.67).

There was a significant interaction effect between age group and species (F_2,87_ = 11.8, p<0.001, $\eta_{p}^{2}=0.21$). Between dog and human expressions, Happy Humans were rated as more positive than Happy Dogs by 4-year-olds (6.4 ± 0.1 vs. 5.4 ± 0.1, respectively; t_27_ = 6.5, p<0.001, *d* = 1.23), by 6-year-olds (6.4 ± 0.1 vs. 5.7 ± 0.2, respectively; t_30_ = 3.6, p<0.01, *d* = 0.64), and by adults (5.7 ± 0.1 vs. 5.1± 0.1, respectively; t_33_ = 4.4, p<0.001, *d* = 0.75). Aggressive Dogs were rated as more positive than Aggressive Humans by 4-year-olds (4.1 ± 0.3 vs. 2.9 ± 0.3, respectively; t_27_ = 5.1, p<0.001, *d* = 0.97) and by 6-year-olds (3.0 ± 0.2 vs. 1.8 ± 0.2, respectively; t_30_ = 5.8, p<0.001, *d* = 1.05), whereas adults rated Aggressive Humans as more positive than Aggressive Dogs (2.6 ± 0.1 vs. 1.9 ± 0.1, respectively; t_33_ = 6.2, p<0.001, *d* = 1.06).

**Fig 3:**
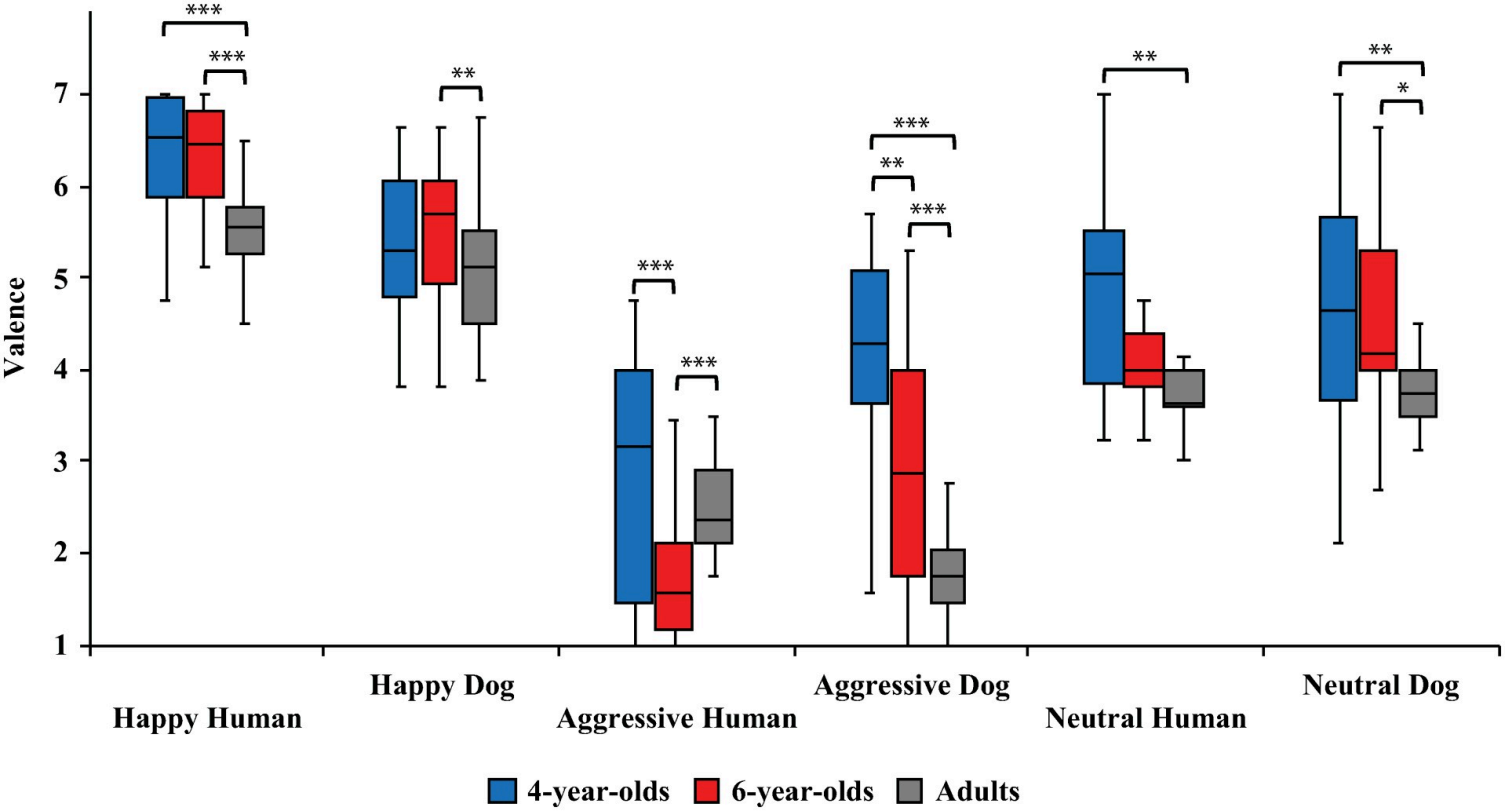
Valence ratings for different image categories (Happy Human, Happy Dog, Aggressive Human, Aggressive Dog, Neutral Human, Neutral Dog) between age groups. Statistically significant differences are represented by asterisks (***p<0.001, **p<0.01 and *p<0.05).

Significant interaction effects were found between species and emotion (F_2,163_ = 49.7, p<0.001, $\eta_{p}^{2}=0.36$), and age group, species, and emotion (F_4,163_ = 14.4, p<0.001, 0.25). Between the age groups, 4-year-olds evaluated Aggressive Human and Dog expressions to be more positive than 6-year-olds (Aggressive Humans: 2.9 ± 0.3 vs. 1.8 ± 0.2, respectively; t_47_ = 3.5, p<0.001, *d* = 0.93; Aggressive Dogs: 4.1 ± 0.3 vs. 3.0 ± 0.2, respectively; t_54_ = 3.3, p<0.01, *d* = 0.88; Fig 3). Compared to adults, 4-year-olds rated more positively Happy Humans (5.7 ± 0.1 vs. 6.4 ± 0.1, respectively; t_51_ = 3.8, p<0.001, *d* = 1.00), Neutral Humans (3.9 ± 0.1 vs. 4.7 ± 0.2, respectively; t_29_ = 3.6, p<0.01 *d* = 1.00), Neutral Dogs (3.9 ± 0.1 vs. 4.8 ± 0.2, respectively; t_42_ = 3.0, p<0.01, *d* = 0.82) and Aggressive Dogs (1.9 ± 0.1 vs. 4.1 ± 0.3, respectively; t_36_ = 8.1, p<0.001, *d* = 2.22). In comparison with adults, 6-year-olds rated more positively Happy Humans (5.7 ± 0.1 vs. 6.4 ± 0.1, respectively; t_63_ = 4.6, p<0.001, *d* = 1.15), Happy Dogs (5.1 ± 0.1 vs. 5.7 ± 0.2., respectively; t_60_ = 2.7, p<0.01, *d* = 0.67), Neutral Dogs (3.9 ± 0.1 vs. 4.5 ± 0.2, respectively; t_54_ = 2.4, p<0.05, *d* = 0.51), and Aggressive Dogs (1.9 ± 0.1 vs. 3.0 ± 0.2 respectively; t_43_ = 4.8, p<0.001, *d* = 1.21). In addition, adults rated Aggressive Humans more positively than 6-year-olds (2.6 ± 0.1 vs. 1.8 ± 0.2, respectively; t_53_ = 3.7, p<0.001, *d* = 0.93).

A significant interaction effect was also found between emotion and age group (F_4,153_ = 5.9, p<0.001, $\eta_{p}^{2}=0.12$), Paired samples t-tests showed that between human categories, all age groups rated Happy Humans as more positive than Neutral or Aggressive Humans (Table 2). Also, Neutral Humans were rated as more positive than Aggressive Humans by all age groups. Between dog categories, all age groups rated Happy Dogs as more positive than Neutral or Aggressive Dogs. Neutral Dogs were rated as more positive than Aggressive Dogs by all age groups.

**Table 2:** The differences in valence ratings (scale 1–7) between human categories and between dog categories in 4-year-olds, 6-year-olds, and adults.

Image category	4-year-olds				6-year-olds				Adults			
	mean (SEM)	t	p	*d*	mean (SEM)	t	p	*d*	mean (SEM)	t	p	*d*
Aggressive vs. Happy Human	2.89 (0.3) 6.36 (0.1)	11.3	0.001***	2.14	1.81 (0.2) 6.35 (0.1)	21.0	0.001***	3.77	2.56 (0.1) 5.71 (0.1)	18.6	0.001***	3.18
Happy vs. Neutral Human	6.36 (0.1) 4.71 (0.2)	7.3	0.001***	1.38	6.35 (0.1) 4.23 (0.1)	14.7	0.001***	2.64	5.71 (0.1) 3.94 (0.1)	15.7	0.001***	2.70
Neutral vs. Aggressive Human	4.71 (0.2) 2.89 (0.3)	6.4	0.001***	1.20	4.23 (0.1) 1.81 (0.2)	11.2	0.001***	2.01	3.94 (0.1) 2.56 (0.1)	10.9	0.001***	1.87
Aggressive vs. Happy Dog	4.11 (0.3) 5.36 (0.1)	4.4	0.001***	0.83	3.00 (0.2) 5.65 (0.2)	11.5	0.001***	2.07	1.88 (0.1) 5.12 (0.1)	18.6	0.001***	3.18
Happy vs. Neutral Dog	5.36 (0.1) 4.75 (0.2)	2.7	0.012*	0.51	5.65 (0.2) 4.48 (0.2)	5.6	0.001***	1.00	5.12 (0.1) 3.94 (0.1)	7.9	0.001***	1.35
Neutral vs. Aggressive Dog	4.75 (0.2) 4.11 (0.3)	2.2	0.039*	0.41	4.48 (0.2) 3.00 (0.2)	5.0	0.001***	0.90	3.94 (0.1) 1.88 (0.1)	13.1	0.001***	2.24

* significant at p < 0.05,

**significant at p < 0.01,

***significant at p < 0.001

### Arousal ratings

A main effect of emotion (F_2,151_ = 133.0, p<0.001, $\eta_{p}^{2}=0.61$, repeated-measures ANOVA) was found, but main effects of age group, species and dog experience were not statistically significant (age group: F_2,87_ = 1.4, p = 0.258, $\eta_{p}^{2}=0.03$; species: F_1,87_ = 1.6, p = 0.207, $\eta_{p}^{2}=0.02$; dog experience: F_1,87_ = 0.9, p = 0.349, $\eta_{p}^{2}=0.01$). However, a significant interaction effect between emotion, species, and age group was found (F_4,169_ = 14.5, p<0.001, $\eta_{p}^{2}=0.25$). Between dog and human expressions, Aggressive Humans were rated higher in arousal than Aggressive Dogs by 4-year-olds (5.0 ± 0.3 vs. 4.3 ± 0.3, respectively; t_27_ = 2.7, p<0.05, *d* = 0.51) and by 6-year-olds (5.9 ± 0.2 vs. 5.0 ± 0.2, respectively; t_30_ = 4.0, p<0.001, *d* = 0.72), whereas Aggressive Dogs were rated higher in arousal than Aggressive Humans in adults (5.7 ± 0.1 vs. 4.9± 0.2, respectively; t_33_ = 4.7, p<0.001, *d* = 0.80). Happy Dogs were rated higher in arousal than Happy Humans in 6-year-olds (3.0 ± 0.2 vs. 2.5± 0.2, respectively; t_30_ = 2.4, p<0.05, *d* = 0.44). Instead, adults rated Happy Humans as higher in arousal than Happy Dogs (4.2 ± 0.1 vs. 3.5 ± 0.2, respectively; t_33_ = 4.4, p<0.001, *d* = 0.75). In addition, adults rated Neutral Dogs higher in arousal than Neutral Humans (3.0 ± 0.2 vs. 2.4 ± 0.2, respectively; t_33_ = 4.4, p<0.001, *d* = 0.75).

**Fig 4:**
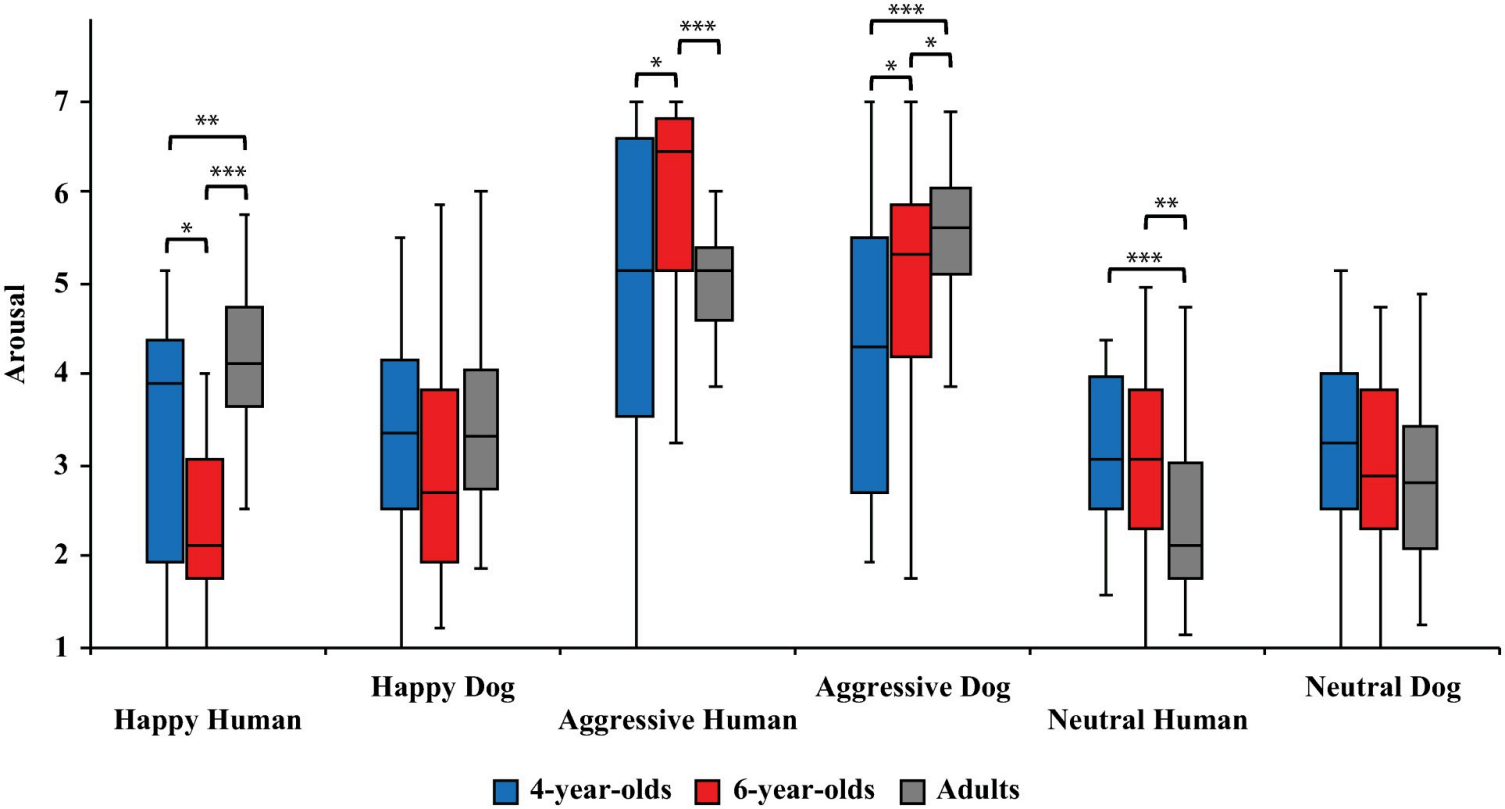
Arousal ratings for different image categories (Happy Human, Happy Dog, Aggressive Human, Aggressive Dog, Neutral Human, Neutral Dog) between age groups. Statistically significant differences are represented by asterisks (***p<0.001, **p<0.01 and *p<0.05).

Between the age groups, 4-year-olds rated Happy Humans higher in arousal than 6-year-olds (3.3 ± 0.3 vs. 2.5 ± 0.2, respectively; t_52_ = 2.5, p<0.05, *d* = 0.65; Fig 4). Furthermore, adults rated the arousal of Happy Humans higher than 4-year-olds (4.2 ± 0.1 vs. 3.3 ± 0.3, respectively; t_40_ = 3.1, p<0.01, *d* = 0.82) or 6-year-olds (4.2 ± 0.1 vs. 2.5 ± 0.2, respectively; t_52_ = 7.2, p<0.001, *d* = 1.8). Neutral Humans were rated higher in arousal by 4-year-olds (3.3 ± 0.1 vs. 2.4 ± 0.2, respectively; t_60_ = 3.8, p<0.001, *d* = 0.94) and by 6-year-olds (3.2 ± 0.2 vs. 2.4 ± 0.2, respectively; t_63_ = 3.1, p<0.01, *d* = 0.78) than by adults. Aggressive Humans (5.9 ± 0.2 vs. 5.0 ± 0.3, respectively; t_50_ = 2.4, p<0.05, *d* = 0.64) and Aggressive Dogs (5.0 ± 0.2 vs. 4.3 ± 0.3, respectively; t_54_ = 2.1, p<0.05, *d* = 0.55) were rated higher in arousal by 6-year-olds than 4-year-olds. In addition, 6-year-olds evaluated Aggressive Humans (5.9 ± 0.2 vs. 4.9 ± 0.2, respectively; t_52_ = 3.8, p<0.001, *d* = 0.95) higher in arousal than adults. Adults rated Aggressive Dogs higher in arousal than 4-year-olds (5.7 ± 0.1 vs. 4.3 ± 0.3, respectively; t_39_ = 4.5, p<0.001, *d* = 1.22) or 6-year-olds (5.7 ± 0.1 vs. 5.0 ± 0.2, respectively; t_49_ = 2.3, p<0.05, *d* = 0.59).

There was also a significant interaction effect between emotion and age group (F_3,151_ = 9.5, p<0.001, $\eta_{p}^{2}=0.18$). Planned comparisons showed that between human categories, all age groups rated Aggressive Humans higher in arousal than Happy or Neutral Humans (Table 3). Also, Neutral Humans were rated higher in arousal than Happy Humans by 6-year-olds, whereas adults rated Happy Humans higher in arousal than Neutral Humans. Between dog categories, all age groups rated Aggressive Dogs higher in arousal than Happy or Neutral Dogs. Also, adults rated Happy Dogs higher in arousal than Neutral Dogs.

**Table 3:** The differences in arousal ratings (scale 1–7) between human categories and between dog categories in 4-year-olds, 6-year-olds, and adults.

Image category	4-year-olds				6-year-olds				Adults			
	mean (SEM)	t	p	*d*	mean (SEM)	t	p	*d*	mean (SEM)	t	p	*d*
Aggressive vs. Happy Human	5.00 (0.3) 3.29 (0.3)	4.4	0.001***	0.84	5.94 (0.2) 2.45 (0.2)	9.6	0.001***	1.73	4.91 (0.1) 4.21 (0.1)	4.4	0.001***	0.75
Happy vs. Neutral Human	3.29 (0.3) 3.25 (0.1)	0.2	0.869	0.03	2.45 (0.2) 3.16 (0.2)	2.7	0.013*	0.48	4.21 (0.1) 2.41 (0.2)	15.2	0.001***	2.61
Neutral vs. Aggressive Human	3.25 (0.1) 5.00 (0.3)	5.8	0.001***	1.10	3.16 (0.2) 5.94 (0.2)	11.4	0.001***	2.04	2.41 (0.2) 4.91 (0.1)	12.3	0.001***	2.11
Aggressive vs. Happy Dog	4.25 (0.3) 3.29 (0.2)	3.3	0.003**	0.62	5.03 (0.2) 3.00 (0.2)	5.8	0.001***	1.05	5.68 (0.1) 3.50 (0.2)	10.0	0.001***	1.72
Happy vs. Neutral Dog	3.29 (0.2) 3.18 (0.2)	0.5	0.621	0.10	3.00 (0.2) 2.87 (0.2)	0.4	0.662	0.08	3.50 (0.2) 2.97 (0.2)	3.3	0.002**	0.57
Neutral vs. Aggressive Dog	3.18 (0.2) 4.25 (0.3)	3.3	0.002**	0.63	2.87 (0.2) 5.03 (0.2)	6.8	0.001***	1.22	2.97 (0.2) 5.68 (0.1)	11.8	0.001***	2.02

* significant at p < 0.05,

**significant at p < 0.01,

***significant at p < 0.001

## Discussion

Generally, all participant groups rated dog and human images in a roughly similar manner, which is in line with the literature reporting commonalities between dog and human facial expression processing [e.g., 59, 60]. Also, children rated the aggressive human and dog faces as less positive and higher in arousal than happy or neutral faces, likewise to adults [25]. Nevertheless, interesting differences between the groups were also detected. More specifically, the current results show that human developmental stage and having daily experience with dogs affect recognizing dog emotional facial expressions and evaluating the valence of the dog and human emotions.

### Development of dog expression recognition is affected by dog experience

In this study, the participant’s age and experience with dogs had an effect on the dog emotion recognition. Adults with dog experience were better at recognizing both Aggressive Dogs and Neutral Dogs than 4-year-olds with dog experience, whereas the performance of experienced 6-year-olds was at the level of adults for both categories. Also, adults without any particular dog experience were better at recognizing Aggressive Dogs than 4-year-olds, but the performance of 6-year-olds were clearly in the middle, not differing from either of the other groups.

The results are in line with previous studies indicating that the ability to recognize dog emotions increases with age [e.g., 14, 16, 61]. Compared to 4-year-old children, adults and 6-year-olds have had the possibility to spend more time around dogs to have an advantage of their general experience with dogs. However, adults rated Aggressive Dogs more often correctly than 4-year-olds regardless of dog experience, and there was no difference between 6-year-olds’ and adults’ performance. These results indicate that the ability to recognize dog emotions, at least some emotions such as aggression, could also be related to the maturation of the emotion processing systems in the brain [e.g., 62, 63]. Results are also in line with a previous study, where 5–6- year-old children were able to recognize aggressive and happy dog expressions even without dog experience [14]. Their sample of 5–6-year-old children falls between many of other studies with children younger and older than those, and the performance of 5–6-year-olds is closer to the performance of 6-year-olds than 4-year-olds in both our study and those of others [16, 29].

In our study, no differences were found between 4-and 6-year-old children’s performance in recognition of human facial expressions. This is important to note, as these age groups did differ in recognizing dog expressions. In addition, no difference was found between any age groups in the recognition of happy dog expressions. These results support previous studies suggesting that happy emotions are recognized with greater accuracy from early on than others such as aggressive or neutral ones [e.g., 40, 64, for a review 65, 66].

### Human interpretation of dog aggression is affected by age and experience

Overall, participants without dog experience rated Aggressive Dogs as more positive than participants with dog experience. Likewise, 4-year-olds evaluated Aggressive Dogs to be more positive than either 6-year-olds or adults, suggesting age has a similar effect on perception of non-conspecific emotional expressions than experience. Furthermore, 4-year-olds regardless of dog experience rated more often correctly Aggressive Humans than Aggressive Dogs, but 6-year-olds with dog experience recognized aggressive dog and human expressions equally well. These results provide further evidence that developmental stage affects human ability to detect dog aggressive expression, and experience with dogs may develop skills to observe dog behavior correctly and to focus on species-typical features [40, 67]. However, in some situations, inexperienced adults have been more successful in identifying some dog expressions than adults with dog experience [13, 28] or there has been no difference between inexperienced and experienced adults and children [15, 23, 37]. Discrepancies between these studies might be due to different study setups and type of stimuli (visual, auditory, audio-visual, facial images, or full body videos) used. Also, different definitions and levels of dog experience may have affected the results. In our study dog experience was characterized with a minimum daily exposure and interaction with a dog—having a dog in the family—whereas in some studies, adults with dog experience have been higher-level experts, for example dog trainers or veterinarians [e.g., 13, 37].

### Age-related effects of theory-of-mind in interpretation of facial expressions

Interestingly, 4-year-olds and 6-year-olds rated Aggressive Humans more often correctly than adults. Adults also rated Aggressive Humans as more positive and lower in arousal than Aggressive Dogs, whereas both child groups rated Aggressive Dogs as more positive and lower in arousal than Aggressive Humans. Possibly, the adults in our sample may have suspected that the humans in the images were not serious, whereas the children lacked such theory-of-mind related suspicion of the actors’ mental state. Theory of Mind (ToM) is a foundational skill allowing children to connect socially with others and to consider others’ perspectives [50]. ToM continues to develop beyond the preschool years as children learn to comprehend nuanced aspects of social cognition [for a review see e.g., 51, 52]. In line with this reasoning, previous studies found that children under the age of six have difficulties detecting pretended anger [68] and that ToM and emotion understanding are closely related in child development [69]. For adults, the threat from non-conspecifics seems to be of higher concern and conspecifics are seen more positively. Happy Humans were also rated as more positive than Happy Dogs in all age groups. The observed pattern of results is also in line previous neuroimaging research showing that 5- to 6-year-olds respond with heightened amygdala activation to human facial expressions compared to adults, especially for angry faces [70].

Our results showed that 4-year-olds and 6-year-olds rated Aggressive Dogs as more positive and lower in arousal than did adults. Because of their age, children have had less possibilities to experience dog facial expressions than adults have, which might have affected to their more positive ratings of Aggressive Dogs. Children’s interaction skills with dogs have shown to develop with age: 2–3-year-olds have more agonistic type interactions than 4–5-year-olds who play more with the dogs [71]. Also, 8- and 10-year-old children and adults are more successful in classifying the contexts of dog barks than 6-year-old children, except aggressive barks toward strangers, which are classified correctly in all groups [23]. In our study, 6-year-olds rated Aggressive Dogs higher in arousal than 4-year-olds, and it might be that because of their developmental level and experience, 6-year-olds were able to evaluate dogs’ threatening expressions more accurately than 4-year-olds. This result is in line with Eretová et al. [16] study using audio-visual stimuli, where 4–5-year-old children showed limited ability to understand dog signals compared to 6–12-year-old children, who successfully recognized dog signals in more than 80% of cases. Another study using video-clips showed that 3–5-year-old children interpreted dogs’ stress signals as signs of happiness or affection [24]. Children’s comments suggest that they anthropomorphized dog’s behavior by trying to find an explanation that would appropriately explain human behavior but was not suitable to explain the dog’s behavior. This might be the case also in our study as 4-year-old children rated aggressive dog images often incorrectly as happy. Aggressive dogs in the images were showing teeth, which made the children think that the dogs were smiling.

### Emotional communication in dogs across sensory modalities

Faces are an important source of information across many species [2; for a review see 72] and an important point of attention from birth [e.g., 73, 74]. Dogs express emotions with their faces especially in communicatory contexts with humans, to the extent that their facial musculature has been evolved from those of wolves [75]. Nevertheless, for dogs and many other species, emotional communication is exhibited throughout their entire body instead of merely the face. In this study, facial pictures were used to obtain knowledge of the specific contribution of facial information for human experience, but in everyday interactions more cues (e.g., bodily gestures, barks) are usually present as an indication of a dog’s emotional state.

The perception of emotional states can also depend on the modality of sensory input, positive states being more understandable when presented visually and negative states acoustically, from the low-frequency growling [37, 76]. According to previous literature, 2–7-year-old children prefer vocal cues over visual cues when categorizing emotional facial expressions of humans [77]. Another study using video clips found that 4–10-year-old children who correctly judged dogs’ behavior as aggressive focused their attention mainly to the sound the dogs were making rather than visual cues [36]. Because of the lack of auditory cues, the aggressive dog images of our study may have been more challenging for children to rate than happy dog images. However, cues are not always correctly recognized and used in interpretations. Humans prefer to focus on facial expressive cues over bodily cues when evaluating dog (and also human) emotional states [27–29]. Without species-specific expertise, humans tend to inspect other species in the same way as humans, although some human and dog facial cues differ from each other (e.g., bared teeth in smiling humans vs. in aggressive dogs) and this might lead into misinterpretation of dog behavior [19, 34, 66, 78, 79].

### Conclusions

Our results provide evidence that both age and daily experience with dogs have an effect on dog emotion recognition and ratings. The results indicate that the ability to correctly interpret dog emotions, especially aggression, increases with age. Limitation in our study is that even though some individuals never had a dog in the family, they might still have dog experience based on for example friends or relatives’ dogs. Another limitation is that the sample size of participants without and with dog experience was unbalanced, which could have affected to the reliability of the results concerning dog experience. However, the relevant effect sizes of the relatively small sample of the study suggest robust differences. In the future, the role of brain structure maturation related to processing of emotional facial expressions, developmental stage in detecting dogs’ expressions, and dog experience in interpreting dog emotions from different cues (e.g., visual and auditory) needs further investigation.
